# Full-Color InGaN/AlGaN Nanowire Micro Light-Emitting Diodes Grown by Molecular Beam Epitaxy: A Promising Candidate for Next Generation Micro Displays

**DOI:** 10.3390/mi10080492

**Published:** 2019-07-24

**Authors:** Ha Quoc Thang Bui, Ravi Teja Velpula, Barsha Jain, Omar Hamed Aref, Hoang-Duy Nguyen, Trupti Ranjan Lenka, Hieu Pham Trung Nguyen

**Affiliations:** 1 Department of Electrical and Computer Engineering, New Jersey Institute of Technology, Newark, NJ 07102, USA; 2 Department of Biomedical Physics, Pham Ngoc Thach University of Medicine, Ho Chi Minh City 700000, Vietnam; 3 Institute of Chemical Technology, Vietnam Academy of Science and Technology, 1 Mac Dinh Chi Street, District 1, Ho Chi Minh City 700000, Vietnam; 4 Department of Electronics & Communication Engineering, National Institute of Technology Silchar, Assam 788010, India

**Keywords:** μLED displays, μLEDs, GaN nanowires, core-shell structure

## Abstract

We have demonstrated full-color and white-color micro light-emitting diodes (μLEDs) using InGaN/AlGaN core-shell nanowire heterostructures, grown on silicon substrate by molecular beam epitaxy. InGaN/AlGaN core-shell nanowire μLED arrays were fabricated with their wavelengths tunable from blue to red by controlling the indium composition in the device active regions. Moreover, our fabricated phosphor-free white-color μLEDs demonstrate strong and highly stable white-light emission with high color rendering index of ~ 94. The μLEDs are in circular shapes with the diameter varying from 30 to 100 μm. Such high-performance μLEDs are perfectly suitable for the next generation of high-resolution micro-display applications.

## 1. Introduction

A display based on inorganic micro light-emitting diodes (μLEDs) has recently been intensively investigated due to its great potential for tech gadgets such as Apple watches, smartphone screens, television screens, billboards, Google glass, and virtual reality devices. Considerable efforts have been poured into this field to bring the novel standard displays to the market [1,2,3,4,5]. The increasing demand for μLED displays in tech screens has received much attention from academia and industry since early last decade. In order to achieve μLEDs displays, it requires several critical stages [6] consisting of making μLEDs, transferring them to a backplane, and precisely controlling each individual LED [7,8,9]. The first essential step is to have the right type of red, green, and blue (RGB) μLEDs satisfactory for the displays. The μLEDs need a long lifespan, superior brightness, high efficiency, and durability. Besides, different approaches for making RGB and white color μLEDs to form a full-color micro-pixel in the μLED displays [10,11,12,13], the monolithic display based on III-nitride nanowire heterostructure μLEDs is promising since it allows more direct control of emission wavelengths of the light-emitting diodes (LEDs). The emission wavelength of nanowire LEDs can be controlled by changing the composition of indium in the InGaN active region. This can be done by adjusting the epitaxial growth conditions including growth temperature and/or In/Ga flux ratios [14,15,16,17]. The energy bandgap of InGaN compounds can be varied from 3.4 eV (GaN) to 0.65 eV (InN) [18], covering the full visible region for display applications. Therefore, GaN based μLEDs are a potential candidate for developing novel micro-LED displays [4].

Conventional planar GaN based LEDs have been used in everyday lighting and automotive headlights. However, their poor operating efficiency and efficiency degradation in the green to red spectrums have limited their potential achievements. The presence of polarization fields [19,20], Auger recombination [21,22], poor hole transport [23], defects/dislocations [24,25], and electron leakage and electron overflow [26,27,28] are the main causes of these above drawbacks. In this regard, nanowire heterostructures have been intensively studied as an alternative candidate for high efficiency light-emitters. Unlike conventional planar structures, nanowires exhibit several distinct advantages, including dramatically reduced strain-induced polarization fields and dislocation densities due to the effective lateral stress relaxation. Moreover, the micron-size nanowire-based LEDs can be much more efficient in heat dissipation due to the reduced current spreading resistance and thereby resulting in increased injection current levels [29,30,31]. Thus, the performance of the nanowire LED is expected to be better than their thin-film counterparts. As compared to organic and inorganic thin-film devices, the brightness, reliability, energy efficiency and moisture resistance of the nanowire LEDs are predicted to be far superior [32]. Therefore, nanowire μLEDs have emerged as a promising candidate for general lighting and display applications. In this context, InGaN/AlGaN nanowire µLEDs in different sizes have been fabricated and characterized. Such nanowire µLEDs exhibit strong and stable emissions from blue to red wavelengths. Moreover, phosphor-free white-color µLEDs have also been demonstrated with highly stable emission.

## 2. Experiment

**Figure 1:**
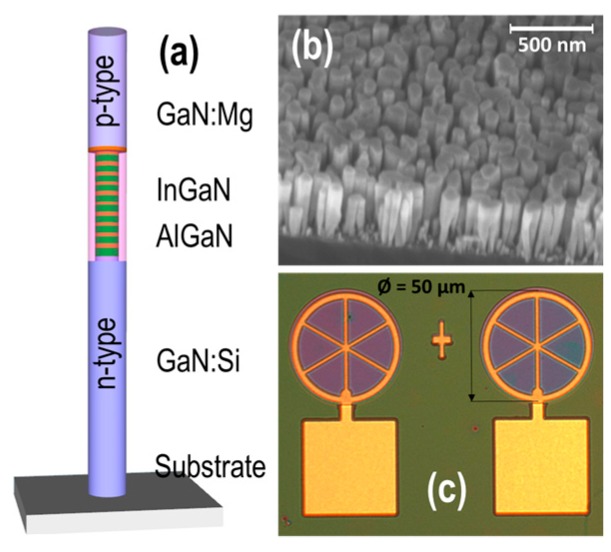
Schematic structure of a nanowire micro light-emitting diodes (μLED) with ten InGaN/AlGaN quantum well heterostructures (**a**); the 45° tilted SEM image of InGaN/AlGaN nanowires on Si substrate (**b**); and optical image of μLEDs and the electrode pads (**c**).

Vertically aligned InGaN/AlGaN core-shell nanowire μLEDs were grown by Veeco Gen II plasma-assisted molecular beam epitaxy (PAMBE) system. Silicon and magnesium dopants were used to grow *n*-GaN and *p*-GaN, respectively. During the epitaxial growth process, the nitrogen flow was kept at 1 sccm and the plasma power was controlled at 350 W. GaN segments were grown at 750 °C, while InGaN in the active region was grown at lower temperatures, in the range of 580–650 °C to enhance the indium incorporation. Figure 1a presents the schematic structure of a single InGaN/AlGaN nanowire on a Si substrate. The nanowire μLEDs consist of GaN:Si grown on a silicon substrate and the GaN:Mg on the top. The ten couples of quantum wells are inserted in the active region. Each quantum well includes a 3 nm InGaN dot and 3 nm AlGaN barrier. During growth of the AlGaN barrier, the AlGaN shell is spontaneously formed, enabling unique core-shell layers [33,34]. The emission color of the μLEDs can be controlled by adjusting the Ga/In flux ratios and the substrate temperature during the MBE growth. For instance, the peak emission wavelength can be shifted from red to blue by gradually increasing the growth temperature of the InGaN active region from 580 °C to 650 °C with ramping rate of 10 °C/min. The nanowire length is controlled by the growth duration. Further information of the core-shell nanowire structures and MBE growth can be found elsewhere [34,35,36,37,38].

Figure 1b shows a scanning electron microscope (SEM) image of InGaN/AlGaN nanowire LEDs taken under a 45° tilted angle. It shows that the nanowires are relatively uniform across the substrate. Figure 1c illustrates the microscopic image of the fabricated μLEDs. The μLED’s emissive window has 50 μm in diameter, which is connected with a square electrode pad. The μLEDs were fabricated using standard photolithography, dry etching and contact metallization techniques, which are described elsewhere [17,37,39,40,41]. During the fabrication process, μLEDs with 30 μm to 100 μm in diameter were defined by standard photolithography. In this paper, the μLEDs with a diameter of 50 μm were chosen for characterization.

## 3. Results and Discussion

**Figure 2:**
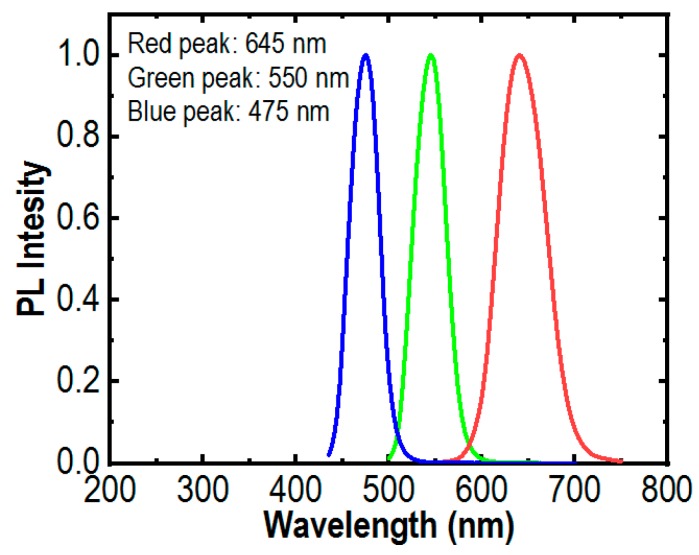
Photoluminescence spectra of the red, green, and blue (RGB) InGaN/AlGaN nanowire µLEDs measured at room temperature.

**Figure 3:**
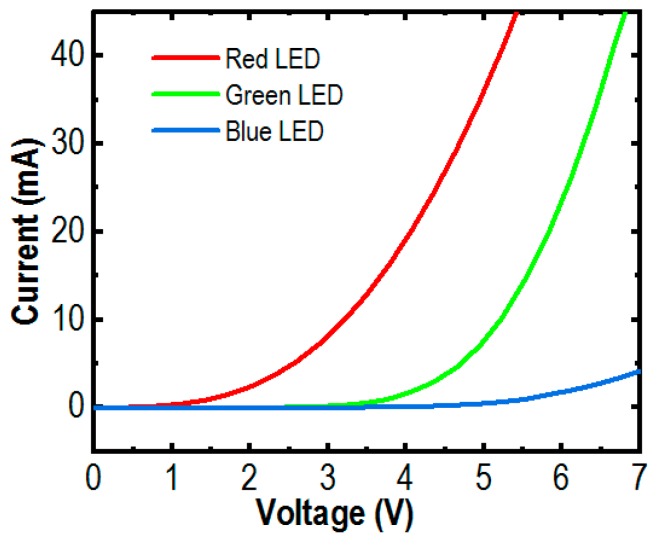
I-V characteristics of the RGB μLEDs are illustrated.

Figure 2 shows the normalized photoluminescence (PL) spectra of the InGaN/AlGaN core-shell nanowires. The measurement was performed at room temperature with a 405 nm laser excitation source. It clearly shows that strong red, green, and blue emissions were recorded at 645 nm, 550 nm, and 475 nm, respectively. The current-voltage characteristics of RGB μLEDs were characterized. The turn-on voltages increase with the decreasing indium composition in the active region of the nanowires. The less indium composition, the higher energy bandgap, makes the light emissions shift toward the blue region. The higher energy bandgap makes turn-on voltages increase [42], as clearly shown in Figure 3. The turn-on voltages of the red, green and blue μLEDs are approximately 1.6 V, 3.5 V, and 4.6 V, respectively. The I-V characteristics also indicate that the devices with a low resistance and good fabrication processes have been achieved.

**Figure 4:**
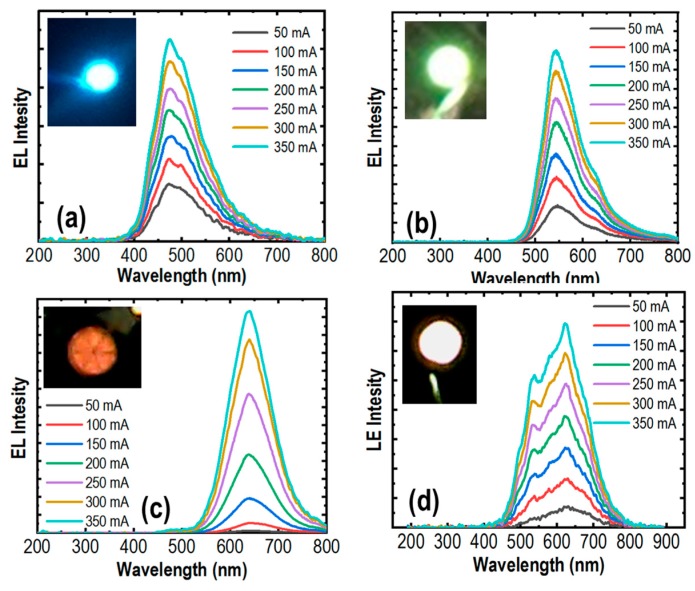
The electroluminescence characteristics of the fabricated blue μLED (**a**), green μLED (**b**), red μLED (**c**), and the white μLED (**d**). The corresponding optical images of these μLEDs are presented in the insets.

**Figure 5:**
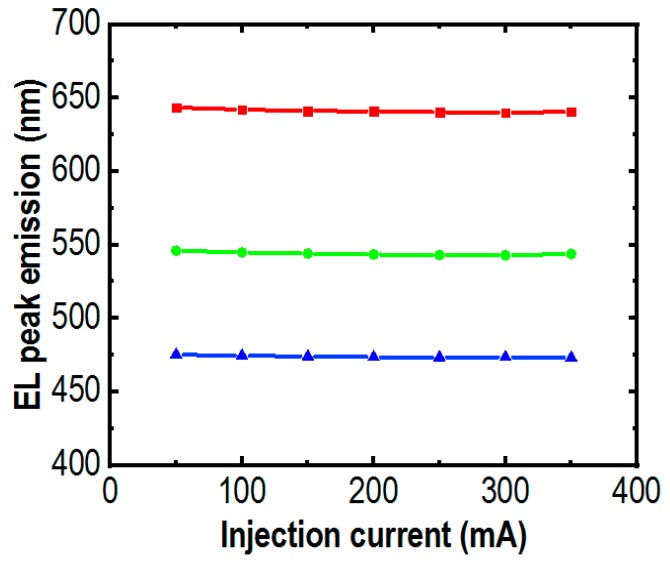
The peak emissions of red, green and blue μLEDs measured under different injection currents from 50 mA to 350 mA.

The electroluminescence (EL) spectra of these μLEDs are presented in Figure 4. The measurements were conducted at room temperature using pulse biasing conditions from 50 mA to 350 mA to reduce the heating effect. Strong red, green, and blue emissions were recorded at 475 nm, 550 nm, and 645 nm for blue, green and red μLEDs, respectively. Illustrated in Figure 4a, highly stable emissions with negligible shift in peak wavelengths were measured for blue μLED with an injection current from 50 mA to 350 mA, attributed to the greatly reduced quantum-confined Stark effect (QCSE) [43] by using nanowire structures. Shown in Figure 4b,c, the blue-shifts of about 1.5 nm were measured for the green and 3 nm for the red μLEDs. These values are significantly smaller than those of planar quantum well LEDs operating in the same spectral regime. Summary of peak wavelength variations of the RGB μLEDs are shown in Figure 5.

III-nitride based planar LEDs operating in the green or longer wavelengths are prone to a blue shift with high driving currents due to QCSE [43]. However, using the nanowire structure, the RGB μLEDs have stable peak wavelengths at 645 nm in red, 550 nm in green, and 475 nm in blue due to the negligible QCSE, meaning that the lattice mismatch induced strain and efficiency droop are negligible [44].

**Figure 6:**
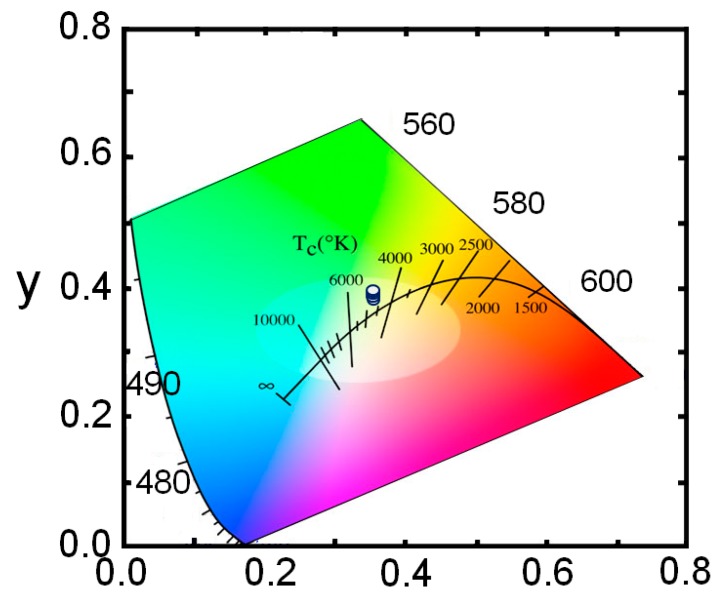
The 1931 Commission International l’Eclairage chromaticity diagram presents stable white light emission characteristics of the phosphor-free white-color InGaN/AlGaN nanowire μLED.

We have also demonstrated white-color μLEDs with strong emission by engineering the emission wavelength of the core-shell nanowire structure. The white light emission covers a long range of the visible spectrum, which is from 450 nm to 750 nm. Moreover, the white-color μLED exhibits a stable emission with a small blue-shift of ~4 nm for injection current from 50 mA to 350 mA. The stable emission characteristics of the phosphor-free white-color μLEDs are further illustrated in the 1931 Commission International l’Eclairage chromaticity diagram in Figure 6. The x and y values are derived to be in the ranges of ~0.351‒0.362 and 0.391‒0.398, respectively. The phosphor-free white-color μLEDs exhibit nearly a neutral white light emission, with correlated color temperature of ~ 4850 K. Additionally, a relatively high color rendering index (CRI) of ~ 94 was measured for this phosphor-free white-color μLED, which is extremely difficult to achieve using planar LED structures. Currently, phosphor-based white LEDs have CRI values in the range of 80–86 [45,46,47,48]. With some special design of phosphor converters, the CRI of these types of white LEDs can be increased up to 90–97 [49,50,51,52,53]. However, the fabrication process of these white LEDs is complex and the device reliability is a major concern. To the best of our knowledge, up to this moment, the CRI value for white-color μLEDs have not yet been reported, possibly due to the complexity of the device fabrication for such μLED devices. In this regard, our phosphor-free full-color and white-color μLEDs are highly desirable for the next generation μLED display technology.

## 4. Conclusions

We have successfully demonstrated μLEDs using InGaN/AlGaN core-shell nanowire heterostructures grown on silicon substrates by MBE. Strong and stable emission from full-color and white-color were recorded from these μLEDs. The color properties of the μLEDs can be optimized by controlling the spectral distribution of the μLEDs. Using nanowire structures, we have achieved phosphor-free white light with unprecedentedly high CRI of ~ 94. The high performance and stable operation of the red, green and blue μLEDs show promise in monolithic μLED displays. Generally, the high cost of current displays is a bottleneck and slows down the market growth. Due to the progressive demand for electronic devices, it is expected to provide lucrative growth opportunities for the micro-display market. In this regard, using the selective area growth approach, RGB subpixel μLEDs can be integrated on the same chip, eliminating the current pick-and-place process, which requires precisely controlling procedures. Therefore, high efficiency, high color rendering properties, and low power consumption μLEDs using GaN nanowire heterostructures are perfectly suitable as an alternative replacement of current display technologies.
